# Retinal Adaptation in Response to Light and Dark Regimes in the Oriental Armyworm *Mythimna separata* (Lepidoptera: Noctuidae)

**DOI:** 10.3390/insects15020135

**Published:** 2024-02-17

**Authors:** Qing-Xiao Chen, Ying Han

**Affiliations:** Laboratory of Insect Evolution and Systematics, College of Horticulture and Plant Protection, Henan University of Science and Technology, Luoyang 471000, China; hanying220320191576@stu.haust.edu.cn

**Keywords:** compound eye, superposition eye, ommatidium, retina, rhabdom, moth, Insecta

## Abstract

**Simple Summary:**

The oriental armyworm, *Mythimna separata* (Walker), is a nocturnal long-distance migratory pest. While searchlight and ground light traps have been widely applied to predict its migration pattern and implement effective pest control measures, the eye structure and adaptation of *M. separata* have received less attention. The moth possesses a pair of superposition eyes, characterized by a clear zone. The retina of *M. separata* shows significant adaptational variations under light and dark conditions: the cross-sectional areas of the ommatidial retinulae and rhabdoms, as well as the rhabdom occupation ratio, within the clear zone are significantly larger under dark adaptation than under light adaptation. The opposite trend is observed beneath the clear zone. These findings indicate remarkable plasticity in the retina of *M. separata* throughout a normal daily cycle, providing valuable information to improve searchlight and ground light trap techniques.

**Abstract:**

The oriental armyworm, *Mythimna separata* (Walker), is a well-known nocturnal migratory pest that relies on its exceptional nocturnal vision for navigation during long-distance flights. In this study, we investigated the ultrastructure of the compound eyes of adult *M. separata* using transmission electron microscopy and quantitatively evaluated adaptational changes in the retina under light and dark conditions. The compound eyes of *M. separata* are superposition eyes with a clear zone. The retina shows remarkable anatomical differences under light and dark adaptation, primarily characterized by distinct patterns of rhabdoms within the clear zone: the rhabdoms are nearly absent under light adaptation, but become more voluminous under dark adaptation. In the distal, middle, and proximal sections of the clear zone, the cross-sectional areas of retinulae and rhabdoms, as well as the rhabdom occupation ratio, are significantly larger under dark adaptation than under light adaptation. Conversely, the opposite trend is observed beneath the clear zone. These results indicate remarkable plasticity in the *M. separata* retina throughout a normal daily cycle, providing a theoretical basis for improving searchlight and ground light trap techniques for the management of this migratory species.

## 1. Introduction

Insects, the most diverse group of arthropods, have evolved to occupy a broad range of temporal niches from full sunlight to dim starlight. Most insects detect ambient light using a pair of compound eyes, which are traditionally categorized into apposition and superposition eyes designed for diurnal and nocturnal activities, respectively [1]. Given the natural day–night rhythm and the resulting fluctuations in environmental light levels experienced by insects, it is crucial for both apposition and superposition eyes to possess adaptational mechanisms to cope with diverse illumination conditions in their habitats [2,3,4].

Superposition compound eye is a specialization of arthropods for vision under dim light [5]. They are anatomically characterized by a pigment-free clear zone that separates the dioptric apparatus from the underlying photoreceptive retina [6]. This structural design allows the superposition of optical signals from multiple ommatidial lenses onto a single photoreceptive rhabdom, significantly enhancing sensitivity in dim light [1]. However, the superposition strategy is less effective in withstanding exposure to intense light, potentially causing severe light-induced retinal damage [7]. Remarkably, the superposition eyes of many insects have evolved many anatomical adaptations to control light flux. One classical adaptational feature involves the dynamic migrations of screening pigment within secondary pigment cells into and away from the clear zone under light and dark conditions, respectively. This phenomenon has been well-documented in many lepidopteran moths [8,9,10,11,12,13,14,15] and other nocturnal insects [16,17,18]. Another anatomical adaptation pertains to the crystalline cone, which has a longer and thinner crystalline cone tract in the light-adapted state than in the dark-adapted state, as observed in some moths [10,19] and other insects [16,17,18,20,21]. However, the retina, the most prominent photoreceptive element, has been poorly studied in terms of light/dark adaptation, with only the translocations of the retinula cell nuclei and changes in the rhabdom occupation ratio (ROR) mentioned in some nocturnal moths [13,14,22].

The oriental armyworm, *Mythimna separata* (Walker), is a seasonal long-distance migratory pest across Asia and Australia, causing substantial yield and economic losses to grain crops annually [23]. As a nocturnal moth, it flies, navigates, and lands under starlight intensities [24]. Given its positive phototaxis, searchlight and ground light traps have been widely applied to predict its north–south migration pattern and implement effective pest control measures in China [25]. In this context, research on the adaptational features of *M. separata* compound eyes, particularly in the retina, under light and dark conditions holds a considerable value for entomologists and agricultural scientists.

In this study, we examined the ultrastructure of the compound eyes of *M. separata*, specifically focusing on the adaptational changes in the retina in the main ventral region of the compound eye without dorsal rim area under short-term light and dark conditions using transmission electron microscopy. The retinal changes were quantified and statistically evaluated by analyzing the cross-sectional areas of the ommatidial retinulae and rhabdoms, as well as ROR.

## 2. Materials and Methods

### 2.1. Insect Collections and Light/Dark Adaptation Experiments

The larvae of *Mythimna separata* (Walker) were collected from the experimental field at Henan University of Science and Technology, Henan Province, China, in June 2018. They were reared under laboratory conditions until reaching the pupal stage. Upon pupation, the male and female pupae were individually placed in separate cages and subjected to a natural illumination cycle (light:dark = 12 h:12 h). After emerging as adults, they were individually maintained under the same light/dark cycle regime for two days before undergoing experimental treatments.

To obtain the compound eyes in the daytime light-adapted state, a group of three males and three females was anesthetized and then decapitated under natural light conditions at 12:00, following approximately 6 h of exposure to indirect sunlight. Conversely, an alternative group of three males and three females was anesthetized and then decapitated under dark conditions at 24:00, after about 6 h in total darkness, to obtain the nighttime dark-adapted compound eyes.

### 2.2. Transmission Electron Microscopy (TEM)

Live light- and dark-adapted adults were anesthetized with diethyl ether. Their compound eyes were excised from the heads and transferred instantly into a fixative mixture of 2.5% glutaraldehyde and 2.0% paraformaldehyde in phosphate-buffered saline (PBS, 0.1 M, pH 7.2) for 12 h at 4 °C. After rinsing with PBS, the samples were fixed with 1% osmium tetroxide (OsO_4_) in PBS (0.2 M, pH 7.2) for 2 h. Following the same rinses, the samples were dehydrated through a graded series of acetone solutions (30%, 50%, 70%, 80%, 90%, and 100%) and infiltrated through mixtures of acetone and Epon 812 resin (3:1, 1:1, and 1:3, *v*/*v*) and pure Epon 812 resin. Finally, the samples were embedded in Epon 812 resin with nadic methyl anhydride as hardener, dodecenyl succinic anhydride as softener, and 2,4,6-tri(dimethylaminomethyl)phenol (DMP-30) as epoxy accelerator. The resin blocks were polymerized at 30 °C for 24 h and 60 °C for 48 h.

We selected the main ventral regions of the compound eyes, excluding the dorsal rim areas, as experimental samples. The ultrathin sections of 70 nm thickness were cut using a diamond knife on the Leica EM UC7 ultramicrotome (Leica, Nussloch, Germany) and placed on 200-mesh formvar-carbon coated grids. The sections on the grids were stained with 2% uranyl acetate for 8 min and 4% lead citrate for 10 min. TEM Micrographs were captured using a JEM-1230 transmission electron microscope (JEOL, Tokyo, Japan) at 80 kV.

### 2.3. Morphometric Analyses

The TEM micrographs were used to quantify several retinal characteristics under light/dark adaptation, including the cross-sectional areas of the ommatidial retinulae and corresponding rhabdoms, as well as the rhabdom occupation ratio to the retinula (ROR) through four distinct sections of the retina: the distal section of the clear zone, the middle section of the clear zone, the proximal section of the clear zone, and the section beneath the clear zone. The symbol “*n*” denoted the number of ommatidia used for measuring various characteristic parameters, which were conducted using ImageJ 1.50i software. All measurement data were independent and did not involve multiple eyes from an individual. To evaluate the “degree of difference” between the four retinal sections under the same adaptational conditions, pair-wise comparisons were conducted using the Tukey Honest Significant Difference (HSD) test (*p* < 0.05). Additionally, the “independent samples” *t*-test was used to examine statistically significant differences (*p* < 0.05) between light- and dark-adapted conditions within the four retinal sections. Mean and standard error were calculated using Predictive Analytics Software Statistics 20.0 (SPSS Inc., Chicago, IL, USA).

## 3. Results

### 3.1. General Anatomy of the Compound Eye

**Figure 1:**
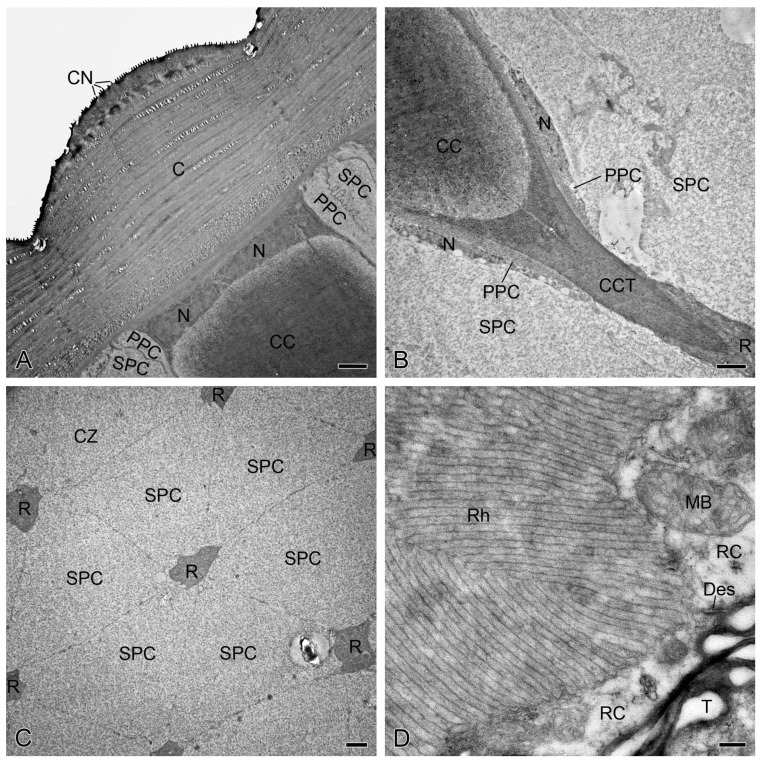
Cellular organizations of the *Mythimna separata* ommatidia, TEM. (**A**) Cornea and crystalline cone. (**B**) Proximal level of the crystalline cone, showing a crystalline cone tract. (**C**) Pigment-free clear zone is composed of secondary pigment cells and separates the ommatidial retinulae from each other. (**D**) Each retinula cell contributes microvilli to a fused rhabdom. C, cornea; CC, crystalline cone; CN, corneal nipple; CZ, clear zone; CCT, crystalline cone tract; Des, desmosome; MB, multivesicular body; N, nucleus; PPC, primary pigment cell; R, retinula; RC, retinula cell; Rh, rhabdom; SPC, secondary pigment cell; T, tracheole. Scale bars: (**A**–**C**) = 2 µm; (**D**) = 200 nm.

The compound eyes of both female and male *M. separata* share a general cellular organization of ommatidia, which each contain a cornea, a eucone crystalline cone, a clear zone, a retinula, a pair of primary pigment cells, six secondary pigment cells, and a basal lamina. The planconvex cornea has a width of approximately 14 μm, and its external surface is covered with tiny nipples (Figure 1A). Beneath the cornea, four cone cells form a eucone crystalline cone and its tract, both of which measure approximately 60 μm and 16 μm in length, respectively, and do not change markedly between light and dark adaptation (Figure 1B). The pigment-free clear zone, approximately 150 μm in width, is formed by secondary pigment cells (Figure 1C) and remains unchanged under light and dark adaptation. Each ommatidial retinula consists of eight retinula cells that contribute their own microvillar rhabdomeres that collectively form a centrally fused rhabdom (Figure 1D). The entire retinula spans approximately 250 μm in length. Seven regular retinula cells are radially arranged in a bundle, extending from the proximal end of the cone to the basal lamina. Within the clear zone, these retinular bundles are spaced far apart (Figure 1C), while below the clear zone, they become almost adjacent to each other, leaving narrow gaps filled by layers of tracheoles (Figure 1D). The eighth retinula cell joins the retinular bundle more than 50 μm distal from the basal lamina.

**Figure 2:**
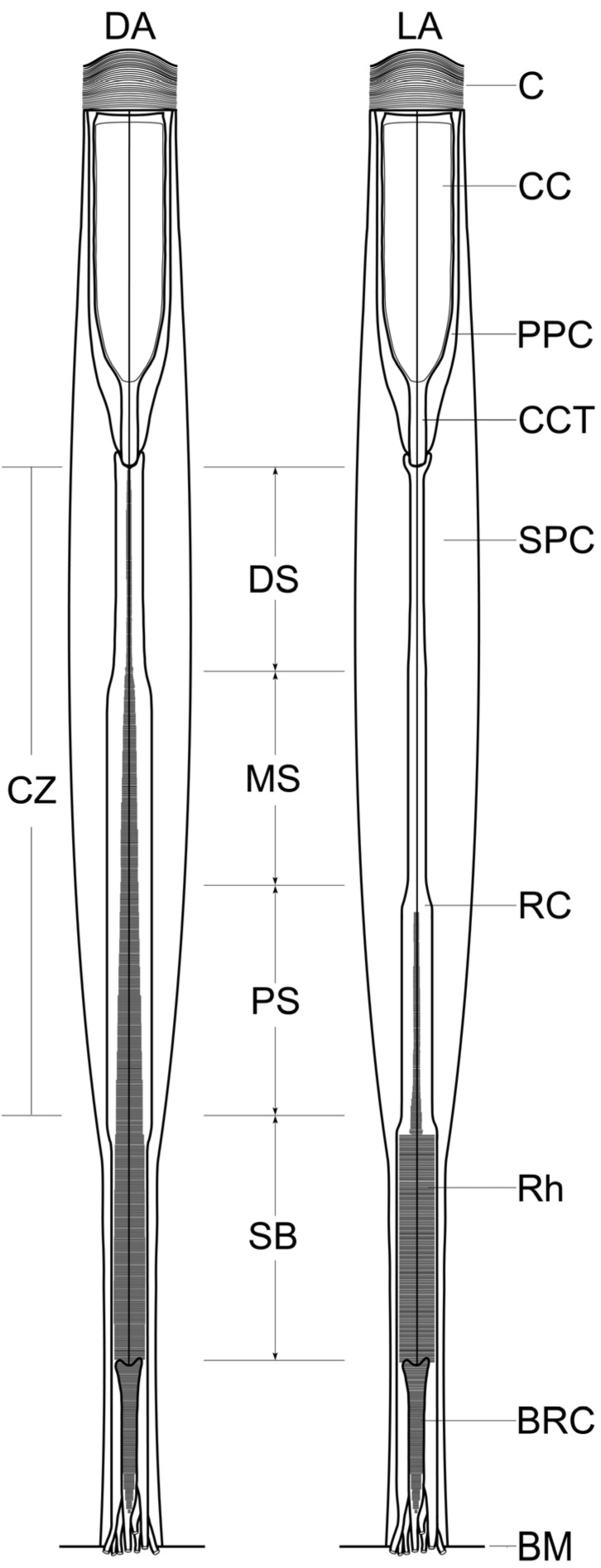
Diagram of dark-adapted (DA) and light-adapted (LA) ommatidia of *M. separata*, showing different adaptational changes in the retina in four different sections: the distal section of the clear zone (DS), the middle section of the clear zone (MS), the proximal section of the clear zone (PS), and the section beneath the clear zone (SB). BM, basal lamina; BRC, basal retinula cell; C, cornea; CC, crystalline cone; CZ, clear zone; CCT, crystalline cone tract; PPC, primary pigment cell; RC, retinula cell; Rh, rhabdom; SPC, secondary pigment cell.

The entire assembly of ommatidial retinulae constitutes the retina, which shows remarkable anatomical changes under light and dark adaptation (Figure 2). For accuracy, these adaptational changes are analyzed in four different sections of the retina: the distal section of the clear zone (approximately 50 μm below the crystalline cone), the middle section of the clear zone (at a depth of roughly 50–100 μm below the cone), the proximal section of the clear zone (at a depth of approximately 100–150 μm below the cone), and the section beneath the clear zone (about 50 μm below the clear zone).

### 3.2. Dark Adaptation in the Retina

**Figure 3:**
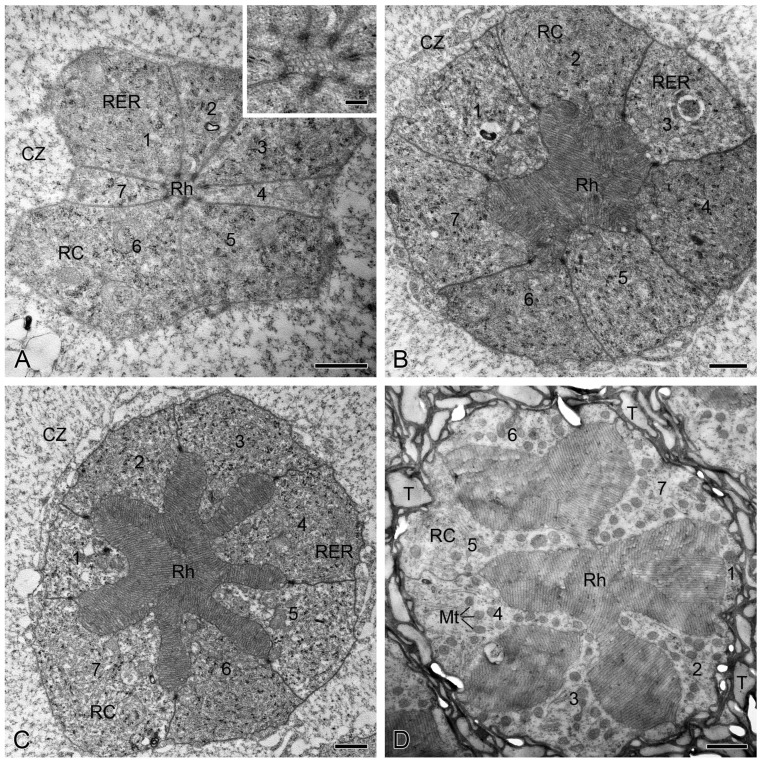
Transverse sections through the ommatidial retinulae of *M. separata* under dark adaptation, TEM. (**A**) Seven retinula cells (1−7) form a tiny rhabdom in the distal section of the clear zone. Inset shows a distal rhabdom. (**B**) Seven retinula cells (1−7) form an irregular rhabdom in the middle section of the clear zone. (**C**) Seven retinula cells (1−7) form a star-shaped rhabdom in the proximal section of the clear zone. (**D**) Seven retinula cells (1−7) form a multi-lobed rhabdom beneath clear zone above the eighth retinula cell. CZ, clear zone; Mt, mitochondrion; RC, retinula cell; RER, rough endoplasmic reticulum; Rh, rhabdom; T, tracheole. Scale bars: (**A**–**D**) = 1 µm; Inset of (**A**) = 250 nm.

**Figure 5:**
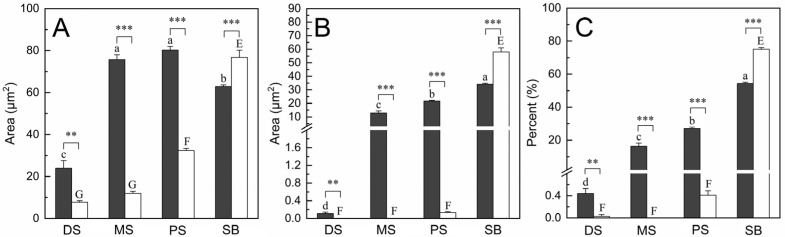
Histograms of the quantitative analyses on the *M. separata* retina across four distinct sections: the distal section of the clear zone (DS), the middle section of the clear zone (MS), the proximal section of the clear zone (PS), and the section beneath the clear zone (SB). The data are presented for both light adaptation (white bars) and dark adaptation (black bars). (**A**) Cross-sectional area of retinulae. (**B**) Cross-sectional area of rhabdoms. (**C**) ROR. Error bars indicate the standard error. Lower-letters (a, b, c, and d) and upper-case letters (E, F, and G) denote significant differences (Tukey HSD, *p* < 0.05) between the four retinal sections under dark and light adaptation, respectively. ** and *** indicate significant differences at *p* < 0.01 and *p* < 0.001 in the independent samples *t*-test, respectively.

In the dark-adapted eyes, each ommatidial retinula traversing the clear zone consists of seven regular retinula cells, which are characterized by abundant rough endoplasmic reticulum in their cytoplasm (Figure 3A–C). Beneath the clear zone, the cytoplasm of the retinula cells is rich in mitochondria (Figure 3D). Within the clear zone, there is a significant increase in the cross-sectional area of retinulae from the distal section, measuring 23.96 ± 3.58 μm^2^ (*n* = 8), to the middle section, measuring 75.71 ± 2.33 μm^2^ (*n* = 20). However, there is no significant difference in the cross-sectional area of retinulae between the middle section and the proximal section, measuring 80.23 ± 1.75 μm^2^ (*n* = 19). Proximally, the cross-sectional area of retinulae decreases to 62.85 ± 0.82 μm^2^ (*n* = 18) in the section beneath the clear zone (Figure 5A).

Beneath the crystalline cone, seven regular retinula cells contribute their rhabdomeres to a centrally fused rhabdom, which is consistently present from the distal section of the clear zone down to the basal lamina. In the distal section of the clear zone, only a few microvilli of the seven retinula cells form the rhabdom (Figure 3A). Proximally, the rhabdom becomes more voluminous and irregular, exhibiting seven triangular rhabdomeres in the middle section of the clear zone (Figure 3B). In the proximal section of the clear zone, the rhabdom further enlarges and shows a star-shaped cross-section with seven wedge-shaped rhabdomeres (Figure 3C). Throughout the section beneath the clear zone, the rhabdom is always multi-lobed in shape (Figure 3D) until the rhabdomere of the eighth retinula cell is involved just above the basal lamina. The cross-sectional area of the rhabdoms exhibits a significant increase from 0.11 ± 0.03 μm^2^ (*n* = 8) in the distal section of the clear zone to 12.82 ± 1.55 μm^2^ (*n* = 20) in the middle section of the clear zone, further to 21.69 ± 0.51 μm^2^ (*n* = 19) in the proximal section of the clear zone, and finally to 34.16 ± 0.73 μm^2^ (*n* = 18) in the section beneath the clear zone (Figure 5B). The statistical differences in ROR among the four sections are consistent with those of the rhabdom cross-sectional area (Figure 5C).

### 3.3. Light Adaptation in the Retina

**Figure 4:**
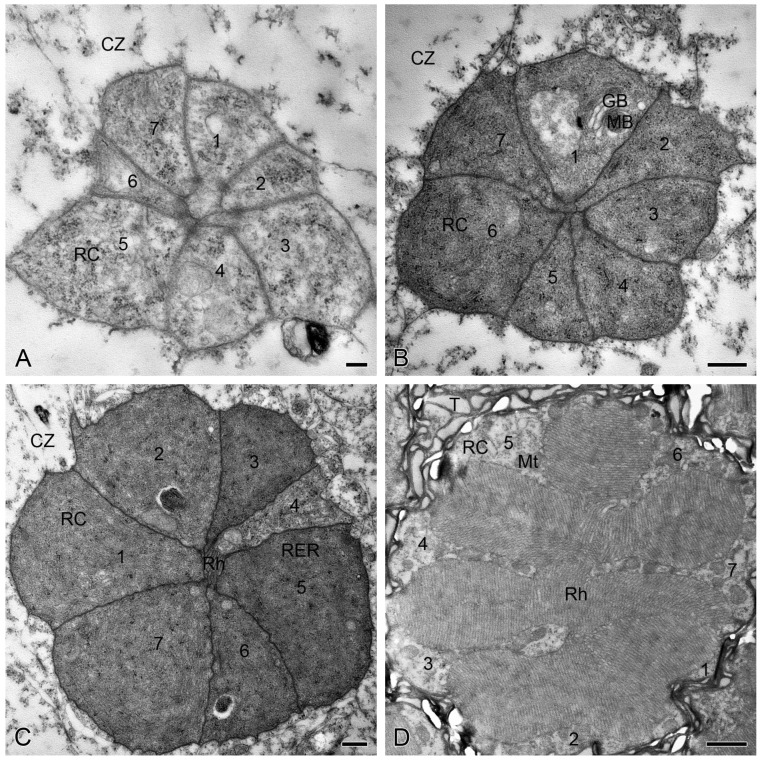
Transverse sections through the ommatidial retinulae of *M. separata* under light adaptation, TEM. (**A**) Seven retinula cell bodies (1−7) are clustered together in the distal section of the clear zone, showing no rhabdom. (**B**) Seven retinula cells (1−7) are present with no rhabdom in the middle section of the clear zone. (**C**) Seven retinula cells (1−7) form a tiny rhabdom in the proximal section of the clear zone. (**D**) A multi-lobed rhabdom occupies almost the entire space of the retinula in the section beneath clear zone. CZ, clear zone; GB, Golgi body; MB, multivesicular body; Mt, mitochondrion; RC, retinula cell; RER, rough endoplasmic reticulum; Rh, rhabdom; T, tracheole. Scale bars: (**A**) = 200 nm; (**B**) and (**C**) = 500 nm; (**D**) = 1 µm.

In the light-adapted eyes, the retinula traversing the clear zone is composed of seven regular retinula cells, which contain numerous vacuoles and multivesicular bodies in their cytoplasm within the clear zone, except for the proximal section (Figure 4A–C). Beneath the clear zone, these retinula cells contain some mitochondria (Figure 4D), which seem to bear relatively fewer than those present in the same section under the dark-adapted state, but we were unable to quantitatively confirm this in the current study. Within the clear zone, there are no significant differences in the cross-sectional area of retinulae between the distal section of 7.76 ± 0.67 μm^2^ (*n* = 8) and the middle section of 11.98 ± 1.00 μm^2^ (*n* = 16). Proximally, the cross-sectional area of retinulae significantly increases to 32.32 ± 1.05 μm^2^ (*n* = 17) in the proximal section of the clear zone and reaches a maximum of 76.76 ± 3.38 μm^2^ (*n* = 18) in the section beneath the clear zone (Figure 5A).

Within the light-adapted clear zone, the rhabdoms are not present in the distal and middle sections until they appear with tiny regularly arranged microvilli in the proximal section of the clear zone (Figure 4A–C). Although the rhabdoms in the proximal section of the clear zone have a cross-sectional area of 0.13 ± 0.02 μm^2^ (*n* = 17) and an ROR of 0.41 ± 0.08 μm^2^ (*n* = 17), these two parameters show no statistical differences among the three sections within the clear zone (Figure 5B,C). Beneath the clear zone, the rhabdoms are multi-lobed and occupy almost the entire space of the retinulae (Figure 4D). The cross-sectional area of the rhabdoms increases significantly to 57.93 ± 3.08 μm^2^ (*n* = 18) (Figure 5B), with a maximum ROR of 75.07 ± 1.02% (*n* = 18) (Figure 5C).

### 3.4. Comparisons between the Dark- and Light-Adapted Retinae

Within the clear zone, the dark-adapted rhabdoms are more voluminous than their light-adapted counterparts (Figure 3 and Figure 4), especially in the distal and middle sections where the rhabdoms are nearly absent under light adaptation (Figure 4A,B). Statistical analyses show that the cross-sectional areas of retinulae and rhabdoms, as well as ROR, in the distal, middle, and proximal sections of the clear zone are significantly larger under dark adaptation than under light adaptation. Conversely, the opposite trend is observed in the section beneath the clear zone (Figure 5).

## 4. Discussion

The compound eyes of the strictly nocturnal *M. separata* are of the superposition type, characterized by a clear zone that separates the dioptric apparatus from the expansive retina. The retina of *M. separata* features eight retinula cells composed of seven regular and one basal retinula cells per ommatidium, forming a fused rhabdom, as is the case in the noctuid *Spodoptera exempta* [11], the lymantriid *Orgyia antiqua* [13], and the arctiine moth *Manulea affineola* [26], all belonging to the superfamily Noctuoidea. The retinal adaptation in response to light and dark regimes primarily involves significant variations in the dimensions of the ommatidial retinula and rhabdom, indicating the remarkable plasticity of the retina throughout a normal daily cycle.

As the photoreceptive sites within the retina, the rhabdoms are the vital focus of interest in our investigations into light and dark adaptation. Through a combination of TEM observations and statistical analyses, a distinct rhabdom pattern emerges within the clear zone: the rhabdoms are nearly absent under light adaptation, but become more voluminous under dark adaptation. Given the nocturnal behavior of *M. separata* [25], the enlargement of rhabdoms likely enhances sensitivity to low light conditions during the night, while their diminution may serve to mitigate the risk of photodamage from intense daylight [4]. However, this case does not hold true in the section beneath the clear zone, where the light-adapted rhabdoms are significantly larger than their dark-adapted counterparts. Unlike *M. separata*, a previous study on the eye adaptation of the lymantriid *O. antiqua* has indicated that, whether during light adaptation or dark adaptation, the rhabdoms are exclusively located beneath the clear zone and are significantly larger in size under dark adaptation than under light adaptation [13]. In the case of the Asian corn borer *Ostrinia furnacalis*, despite the lack of statistically analyzed evidence, observations through TEM have revealed that the rhabdoms are consistently present within the clear zone under both light and dark adaptation, and appear to be more prominent beneath the clear zone during dark adaptation [22]. While relatively little research has previously explored the adaptational changes in rhabdoms in insect superposition eyes, we speculated, based on the aforementioned cases, that the distribution patterns and adaptational changes of rhabdoms might be highly diverse, potentially correlating not only with the state of illumination, but also with certain endogenous demands, such as circadian rhythms and endocrine regulation for migration.

Clearly consistent with the fluctuation trends in rhabdom volume are those in the volume of the corresponding ommatidial retinula through the entire retina of *M. separata*. Specifically, the retinula exhibits a significantly larger area under dark adaptation than under light adaptation within the clear zone, whereas the reverse holds true beneath the clear zone. Much less is previously documented about the changes in the size of retinula bundles during different adaptation’ only in female *O. antiqua* has there been a brief mention that the retinula bundles expand to occupy a larger space during dark adaptation, owing to screening pigment granules leaving the clear zone [13]. In contrast, in the superposition eyes of nocturnal moths [14,19], beetle [20,21], and lacewing [17], the adaptational changes in retinula bundles have primarily been documented as changes in the position of distal retinula cell bodies connecting with the crystalline cone, i.e., the distal retinula cell bodies move up and down as the crystalline cone tracts shorten and lengthen during dark and light adaptation, respectively. These retinal movements often coincide with the rearrangement of their nuclei and rhabdomeres [10,17,19]. Given that our research has exclusively focused on the retina of compound eyes without the crystalline cone, it cannot be determined whether the observed changes in the size of retinulae are linked to their longitudinal migration.

The extent of retinal changes is considered to be influenced by the duration of illumination and circadian rhythmicity [2]. Recent research using optical coherence tomography has demonstrated that it could take approximately 30 min for a moth’s eye to undergo a complete transition between the light- and dark-adapted states [27]. In our study, the retina of *M. separata* exposed to approximately six hours of either light ending at noon or darkness terminating at midnight is likely to induce maximum adaptational changes. However, it is important to acknowledge that these extreme states may not be sustained for as prolonged a period in the wild as observed under these experimental conditions. This is due to the natural variation in illumination throughout the light and dark phases of the day. Then, the next step is to investigate adaptational changes in the retina when subjected to daytime darkness and nighttime illumination, which would be quite intriguing.

Furthermore, our present study has revealed no sexual differences in the eye structure and the retinal adaptation in *M. separata*, different from the moths with distinct sexual dimorphism, where variations in eye structure and adaptation between the sexes are observed [13,15]. However, the possibility that the retinal adaptation may vary across different stages of adult *M. separata* cannot be overlooked. Given that our study focused on recently emerged adults, further research is required to explore how adaptational changes in the retina occur in individuals before and after mating, before and after oviposition, as well as in individuals nearing the end of their lifespan.

## Data Availability

The data presented in this study are available on request from the corresponding author.
